# Baricitinib, a JAK-STAT Inhibitor, Reduces the Cellular Toxicity of the Farnesyltransferase Inhibitor Lonafarnib in Progeria Cells

**DOI:** 10.3390/ijms22147474

**Published:** 2021-07-12

**Authors:** Rouven Arnold, Elena Vehns, Hannah Randl, Karima Djabali

**Affiliations:** Epigenetics of Aging, Department of Dermatology and Allergy, TUM School of Medicine, Technical University of Munich (TUM), 85748 Garching, Germany; rouven.arnold@tum.de (R.A.); elena.vehns@tum.de (E.V.); hannah.randl@tum.de (H.R.)

**Keywords:** progerin, lamin, baricitinib, farnesyltransferase inhibitor, replicative senescence, JAK-STAT, inflammation, age-related disease, progeria

## Abstract

Hutchinson–Gilford progeria syndrome (HGPS) is an ultra-rare multisystem premature aging disorder that leads to early death (mean age of 14.7 years) due to myocardial infarction or stroke. Most cases have a de novo point mutation at position G608G within exon 11 of the *LMNA* gene. This mutation leads to the production of a permanently farnesylated truncated prelamin A protein called “progerin” that is toxic to the cells. Recently, farnesyltransferase inhibitor (FTI) lonafarnib has been approved by the FDA for the treatment of patients with HGPS. While lonafarnib treatment irrefutably ameliorates HGPS disease, it is however not a cure. FTI has been shown to cause several cellular side effects, including genomic instability as well as binucleated and donut-shaped nuclei. We report that, in addition to these cellular stresses, FTI caused an increased frequency of cytosolic DNA fragment formation. These extranuclear DNA fragments colocalized with cGAs and activated the cGAS-STING-STAT1 signaling axis, upregulating the expression of proinflammatory cytokines in FTI-treated human HGPS fibroblasts. Treatment with lonafarnib and baricitinib, a JAK-STAT inhibitor, not only prevented the activation of the cGAS STING-STAT1 pathway, but also improved the overall HGPS cellular homeostasis. These ameliorations included progerin levels, nuclear shape, proteostasis, cellular ATP, proliferation, and the reduction of cellular inflammation and senescence. Thus, we suggest that combining lonafarnib with baricitinib might provide an opportunity to reduce FTI cellular toxicity and ameliorate HGPS symptoms further than lonafarnib alone.

## 1. Introduction

Hutchinson–Gilford progeria syndrome (HGPS: OMIM #176670) is a rare human genetic disease with an incidence of only 1 in 20 million births [1]. The main clinical features are accelerated aging and the development of several pathologies, such as atherosclerosis, arthritis, lipodystrophy, and alopecia, with the early death of affected children in their teens [2,3]. Symptoms and molecular mechanisms underlying HGPS overlap greatly with normal aging, making it a good model for gaining more knowledge on age-associated diseases in general and possibly discovering new therapies [2,4]. In the vast majority of cases the genetic cause of HGPS is linked to a de novo heterogeneous G608G point mutation in the lamin A/C (*LMNA*) gene (c.1824C > T; GGC > GCT), leading to the production of a truncated prelamin A protein, called progerin [5,6]. Incorporated into the nuclear lamina, progerin leads to disruption of the nuclear membrane architecture and triggers cellular alterations, including abnormal gene expression, DNA damage, mitochondrial dysfunction, and premature senescence (SNS) [7,8]. In normal cells, the *LMNA* gene translates into two lamin isoforms: prelamin A and lamin C. While lamin C is already a mature protein, prelamin A needs to be further processed to produce mature lamin A [9]. This process involves binding of a farnesyl group to the cysteine at the C-terminal CAAX motif of prelamin A, which is then proteolytically removed by either Ras converting enzyme 1 (RCE1) or the zinc metallopeptidase STE24 (ZMPSTE24). Subsequently, the terminal cysteine is then carboxy-methylated and an internal proteolytic cleavage occurs, thereby removing the last 15 coding amino acids that contain the two modifications by ZMSPTE24 [10]. Progerin still retains the CAAX motif but lacks the endoproteolytic cleavage site, resulting in a permanently farnesylated protein that remains attached to the nuclear envelope [11].

Several therapeutic strategies have been investigated to improve the clinical status and lifespan of patients with HGPS, but there is still no cure [4]. The first clinical approach for HGPS is to prevent the farnesylation of prelamin A [12]. This strategy results in less progerin being incorporated into the nuclear envelope, consequently ameliorating the nuclear envelope morphology [13]. Several studies using farnesyltransferase inhibitors (FTIs) have shown improvements at the cellular and organismal levels in HGPS models [11,12,13,14,15,16,17,18,19,20,21,22,23,24,25,26,27,28,29,30,31,32]. In different transiently transfected human cancer or kidney cells as well as mouse cultures and human HGPS fibroblasts, blocking farnesylation restored nuclear shape, reduced nuclear blebbing, and decreased progerin levels [12,14,15,16,17,18].

Further indirect cellular improvements by FTI have been described, such as reduced osteogenic differentiation of HGPS-induced pluripotent stem cells [19], enhanced DNA damage response [20,21], amelioration of the mitochondrial function [22], improved autophagy [23], and correction of the chromosome positioning in interphase HGPS cells [24]. However, aside from the inhibition of progerin farnesylation, little is known about the direct effect of FTI on HGPS cells. Prenylation is an important posttranslational mechanism in the cell, which influences many farnesyltransferase substrates, including prelamin A, lamin B, Rheb, Ras, and other proteins [33,34]. In this regard, different deleterious effects of FTI have been observed, such as induction of binucleated cells and abnormal donut-shaped nuclei [25], delocalization of A- and B-type lamins away from the inner nuclear envelope [26,35], and a reduction in the proliferation rate [23]. However, FTI treatment of several HGPS mouse models has shown substantial improvements, including lifespan extension, improvement in bone mineralization and body weight [27,28,29] and reduction in cardiovascular defects [30]. In 2007, the first clinical trial of patients with HGPS was initiated using the FTI lonafarnib (ClinicalTrials.gov, accessed on 11 July 2021, NCT00425607). In 2012, the results demonstrated that lonafarnib ameliorated some clinical features of patients with HGPS, including the vital cardiovascular system [31]. In brief, patients showed prolonged mean survival time by 1.6 year, a modest increase in weight, reduction in vascular stiffness, increased skeletal rigidity, and some amelioration in sensorineural hearing and bone mineral density [11,31,32]. These positive clinical outcomes underlie the approval of the Food and Drug Administration (FDA) to prescribe lonafarnib for the treatment of HGPS [36]. In spite of these improvements, lonafarnib is not a cure for children with HGPS; therefore, further research is needed to test new candidate drugs in combination with lonafarnib to permit further amelioration of HGPS conditions.

Emerging evidence indicates that chronic inflammation develops with age, as reported in numerous age-related diseases, including arthritis and vascular disease [37,38]. In this regard, the JAK/STAT signaling pathway is involved in many cellular processes such as cellular immunity, cell death, and tumor formation, which could trigger inflammatory response if not regulated properly [39]. Recently, we demonstrated that baricitinib (Bar), a potent JAK1/2 inhibitor [40], and FDA approved for rheumatoid arthritis [41], could rescue HGPS cellular phenotypic changes [7]. We also showed that the JAK/STAT pathway was overactivated in HGPS cells and demonstrated that Bar treatment restored several cellular malfunctions [7]. Taken together, we found that Bar restored cellular homeostasis, delayed SNS, decreased proinflammatory markers, and reduced progerin levels [7]. Supporting our findings, a study using Ruxolitinib as an alternative JAK1/2 inhibitor to treat Zmpste24^−/−^ mice showed that it could decrease the incidence of bone fractures, elevate mineral bone density, improve grip strength, and increase the lifespan of these progeroid mice [42].

To advance the efficacy of treatment for patients with HGPS, it is evident that a single drug regimen cannot reverse all cellular and tissue defects that characterize HGPS disease. This will require combining drugs to target distinct cellular processes, while still acting synergistically and complementarily to permit lower dosage, and thereby reduce toxicity and side effects inherent to each drug. For instance, high doses of Bar cause excessive blockade of immune networks and result in opportunistic infections such as herpes zoster, herpes simplex, and upper respiratory tract infections [43]. Moreover, JAK inhibitors cause a decrease in neutrophil count [44]. In the case of FTI, the side effects include mild diarrhea, fatigue, nausea, vomiting, anorexia, and depletion of serum hemoglobin, all of which seem preventable by low FTI dosages [31].

In this study, we tested whether combining the JAK1/2 STAT1/3 inhibitor Bar, to reduce the SNS-associated secretory phenotype (SASP), with lonafarnib, to prevent farnesylation of progerin, will amend HGPS cellular alterations more than each drug alone. To monitor the efficacy of this therapeutic strategy, we examined several cellular functions, including proliferation, SNS, autophagy, proteasomal activity, mitochondrial function, and key pathways that are altered in HGPS cells. Our findings indicate that Bar reduces the negative cellular defects associated with FTI, and their co-administration ameliorates nuclear morphology, progerin levels, and several HGPS cellular malfunctions.

## 2. Results

### 2.1. FTI with Bar Combination Treatment Inhibits JAK-STAT Signaling and Improves Proliferative Rate of HGPS Fibroblasts

Bar (LY3009104) is a small molecule that selectively and reversibly inhibits the Janus kinases 1 and 2 (JAK1 and JAK2), as well as blocks the activation of the STAT family of transcription factors [40]. JAKs are tyrosine kinases that transduce cytokine-mediated signals, including type I interferons, interleukins, growth factors, and other extracellular signaling proteins [45]. FTI (lonafarnib) prevents the transfer of a farnesyl moiety to the cysteine in proteins harboring a carboxyl-terminal CAAX motif and has been shown to block progerin farnesylation [15]. FTI treatment reverses the nuclear envelope abnormalities in HGPS cells [12,14]. Mechanistically, Bar and FTI act on distinct cellular processes, and their combination was expected to synergistically improve the cellular phenotype of HGPS.

**Figure 1:**
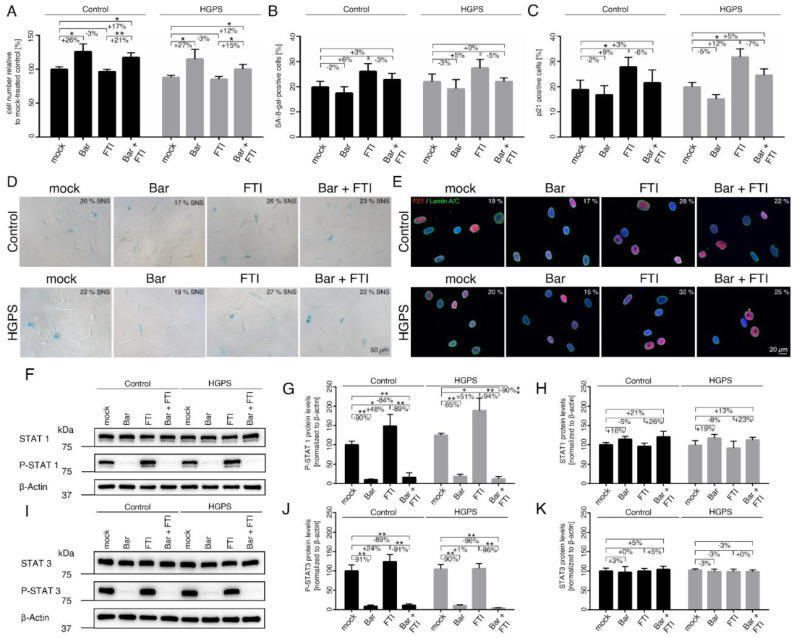
Replicative senescence of control and HGPS fibroblasts after treatment with baricitinib and FTI. (**A**) Cell numbers relative to mock-treated control cells. All cultures were started with ~15% senescence. Cells were either treated with vehicle (DMSO), Bar (1 µM), FTI (0.025 µM), or combined drugs for a period of nine days. (**B**) Graph shows the percentage of SA-ß-gal-positive cells measured after indicated treatments. (**C**) Percentage of cells positive for p21 after indicated treatment. (**D**) Representative images of SA-ß-gal-positive cells in treated cultures. Scale bar: 50 µm. (**E**) Representative immunofluorescence images of control GM01651C and HGPS HGADFN127 fibroblasts after the indicated treatment. Antibodies against p21 (red) and lamin A/C (green) were used, and DNA was stained with DAPI. Fluorescence images were taken at 40× magnification. Scale bar: 20 µm. (**F**,**I**) Representative images of western blots for STAT1, P-STAT1, STAT3 and P-STAT3. Quantification of P-STAT1 (**G**), STAT1 (**H**), P-STAT3 (**J**), and STAT3 (**K**). Graphs show mean ± SD. Representative images are shown (*n* = 3; * *p* < 0.05, ** *p* < 0.01).

Previously, we reported that the JAK/STAT pathway was overactivated during replicative SNS, and treatment with 1 µM Bar was sufficient to inhibit this pathway in control (Ctrl) and HGPS cultures [7]. In another study, we determined that 0.025 µM of lonafarnib effectively reduced progerin levels in HGPS cells [23]. Therefore, we selected 1 µM Bar and 0.025 µM of FTI as dosages for all experiments in this study. FTI, Bar, and combined treatments were carried out for a period of nine days on control (GM01651C, GM01652C) and HGPS (HGADFN003, HGADFN127) primary fibroblast cultures that had reached 15% SNS (Figure 1).

The growth rates and SNS indexes were determined after all treatments as indicated (Figure 1A–E). While Bar treatment increased proliferation by ~25% in both control and HGPS cultures, FTI-treated cells maintained a similar growth rate as mock-treated cells (Figure 1A). Combination treatment increased proliferation by 17% in control and 12% in HGPS cultures compared to mock-counterparts but remained lower than with Bar treatment alone (Figure 1A). On day nine, the SNS index of the cultures treated with the different regimens showed no significant changes relative to their mock counterparts (Figure 1B,D). Because ß-gal associated SNS is a late marker of SNS and may not reflect the real number of SNS-affected cells, we also scored cells positive for p21, an early marker of SNS (Figure 1C,E). In accordance, with the growth rates, the percentage of p21 positive cells in the Bar-treated cultures was slightly reduced (Ctrl: −2% ns, HGPS: −5% ns), while in cultures treated with FTI, the percentage of p21 positive cells was higher (Ctrl: +9%, HGPS: +12%) relative to the other regimens (Figure 1C). In combination-treated cultures, the number of p21 positive cells was similar to that of mock cells (Figure 1C). These findings indicate that Bar prevented cellular SNS caused by FTI treatment.

Since Bar is an inhibitor of the JAK-STAT pathway, we assessed the status of STAT1/3 and its phosphorylated forms (p-STAT1/3) in control and HGPS cultures. Cultures that had reached 15% SNS were treated for nine days (Figure 1F–H). The STAT1 levels tended to increase in Bar-treated control and HGPS cells with or without FTI, though not significant (Figure 1F,H). STAT1 levels in FTI-treated cells remained unchanged (Figure 1H). Furthermore, STAT3 levels remained constant in all regimens (Figure 1I,K). As expected, the levels of p-STAT1 and p-STAT3 were significantly decreased in both Bar-with or without FTI-treated cells (Figure 1F–K). In strong contrast, treatment with FTI alone induced a sharp increase in the levels of p-STAT1 in both cell types (Ctrl: +48%, HGPS: +51%) (Figure 1G). However, the levels of p-STAT3 in FTI-treated cultures remained constant in both control and HGPS cells compared to mock-treated counterparts. Collectively, these findings indicate that FTI induces the activation of STAT1 signaling.

### 2.2. FTI Treatment Induces Donut-Shaped Nuclei, Cytoplasmic DNA Foci, and Cyclic GMP-AMP Synthase (cGAS) Activation

To dissect the mechanism underlying STAT1 activation in cells treated with FTI, we first re-examined the effect of FTI on genomic instability, as reported previously [25]. As already described, donut-shaped nuclei occur after FTI treatment due to a centrosome separation defect in mitotic cells [25], in addition to an accumulation of prelamin A, mislocalization of lamin B2 to the nucleoplasm, and decreased lamin B1 levels at the nuclear envelope [35].

**Figure 2:**
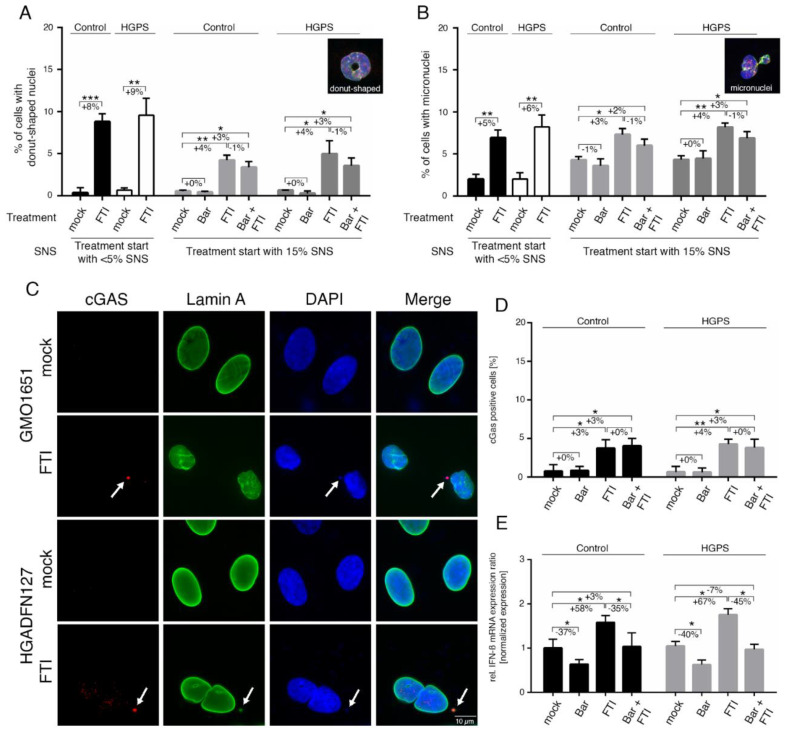
FTI treatment activates the cGAS-STING pathway. (**A**) Determination of the frequency of donut-shaped nuclei with indicated treatment and senescence (SNS). Cells were either treated with vehicle (DMSO), Bar (1 µM), FTI (0.025 µM), or combined drugs for a period of nine days. (**B**) Determination of the frequency of micronuclei at indicated treatment and senescence after a period of nine days. (**C**) Representative immunofluorescence images of an HGPS (HGADFN127) fibroblast cell strain treated for nine days as indicated. Antibodies against lamin A (green) and cGAS (red) were used, counterstained with DAPI. Fluorescence images were taken at a 60× magnification. Scale bar: 10 µm. (**D**) Percentage of cells positive for cGAS after indicated treatment. (**E**) Quantitative real-time PCR analysis of IFN-β in cells treated as indicated. Relative expression was normalized to expression of GAPDH. Graphs show mean ± SD. Representative images are shown (*n* = 3; * *p* < 0.05, ** *p* < 0.01, *** *p* < 0.001).

These FTI-induced defects occurred in both the normal and HGPS cells. Therefore, we re-investigated the occurrence of donut-shaped nuclei, micronuclei, and cytoplasmic chromatin foci that could result from chromosome segregation defects or altered cytokinesis upon FTI treatment (Figure 2A–D).

In accordance with these previous studies, an increased number of donut-shaped nuclei was observed in FTI—with or without Bar-treated cultures (Figure 2A). Hence, the proportion of cells that exhibited the presence of micronuclei was also elevated by FTI and combination treatment (Figure 2B). As these defects must occur during cell division, we examined early culture passages (~5% SNS), which divide more frequently than older cells (Figure 2A,B). Indeed, the frequency of donut-shaped nuclei and micronuclei in FTI-treated cultures was increased (Figure 2A,B). The presence of cytoplasmic DNA foci suggested that the cytosolic DNA-sensing cGAS-STING pathway of the innate immune system was activated [46,47]. To validate this assumption, we scored the number of cells harboring cytosolic DNA foci that were positive for cGAS by immunohistochemistry (Figure 2C,D). The percentage of cells with cGAS/DNA-positive cytosolic foci was increased in FTI-treated cells (Ctrl: +3%, HGPS: +4%), (Figure 2D). Combination treatment induced a similar increase as FTI alone (Ctrl: +3%, HGPS: +3%) (Figure 2D). These findings indicate that FTI causes the release of chromatin fragments into the cytoplasm, leading to the activation of the cGAS-STING pathway, a regulator of the expression of inflammatory genes such as type I interferons (IFN) [47].

Type I IFNs are known to activate JAK 1 kinase and tyrosine kinase 2 (TYK2), which in turn phosphorylate STAT1 and STAT2 [48]. However, IFNs are expressed in a cell-type-specific manner, and IFN-β can be detected in fibroblasts [48]. In accordance with our assumption, IFN-β expression levels were increased in both control and HGPS fibroblast cultures treated with FTI (Ctrl: +58%, HGPS: +67%), (Figure 2E). In contrast, the levels of IFN-β were reduced in cultures treated with Bar alone (Ctrl: −37%, HGPS: −40%), while the drug combination treatment normalized IFN-β mRNA levels to the levels observed in mock-treated cells (Figure 2E). Collectively, these findings identified an additional FTI cellular side effect, which is the activation of the cGAS-STING-STAT1 signaling axis, as evidenced by the enhanced p-STAT1 levels in FTI-treated cultures.

### 2.3. FTI with Bar Combination Treatment Reduced the Expression of Proinflammatory Cytokines

**Figure 3:**
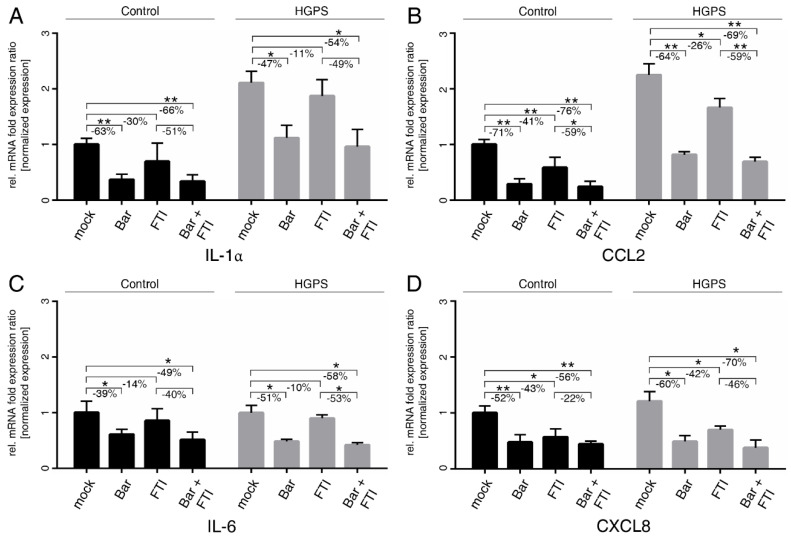
SASP factors are blunted by treatment with Bar and FTI. (**A**–**D**) Quantitative real-time PCR analysis of IL-1α, CCL2, IL-6, and CXCL8. Cultures at 15%SNS were either treated with vehicle (DMSO), Bar (1 µM), FTI (0.025 µM), or combined drugs for a period of nine days. Relative expression was normalized to expression of GAPDH. Graphs show mean ± SD (*n* = 3; * *p* < 0.05, ** *p* < 0.01).

Replicative SNS is characterized by functional and secretory changes, which among others occur during long-term cultures [49]. Previously, we reported that proinflammatory factors such as interleukin 1α (IL-1α), IL-6. Chemokine ligand 2 (CCL2) and IL8/CXCL8 (typical SASP factors) are upregulated during replicative SNS in control and HGPS cultures [7]. Therefore, we determined their expression profiles upon treatment with FTI, Bar, and combination treatment in normal and HGPS cultures (Figure 3).

Bar with or without FTI treatments efficiently reduced the levels of IL-1α and CCL2 in both cell types and normalized their levels in HGPS cells to mock-treated control cells (Figure 3A,B). IL-6 and CXCL8 mRNA levels were reduced by Bar with or without FTI treatments in both cell types (Figure 3C,D). Overall, FTI treatment alone showed a tendency to reduce the mRNA levels of all tested cytokines, but their suppression was lower and not always significant than that observed with Bar treatment alone (Figure 3). However, the overall reduction in cytokine mRNA levels with combination treatment was higher than that in the Bar treatment alone. These findings indicated that combination treatment exerted a positive and synergistic effect on reducing the expression of these cytokines (Figure 3). Collectively, FTI and Bar combination treatment reduced the expression of proinflammatory factors, known to be expressed by SNS cells and to drive inflammation.

### 2.4. FTI with Bar Combination Treatment Enhanced Progerin Clearance and Restored Nuclear Morphology

**Figure 4:**
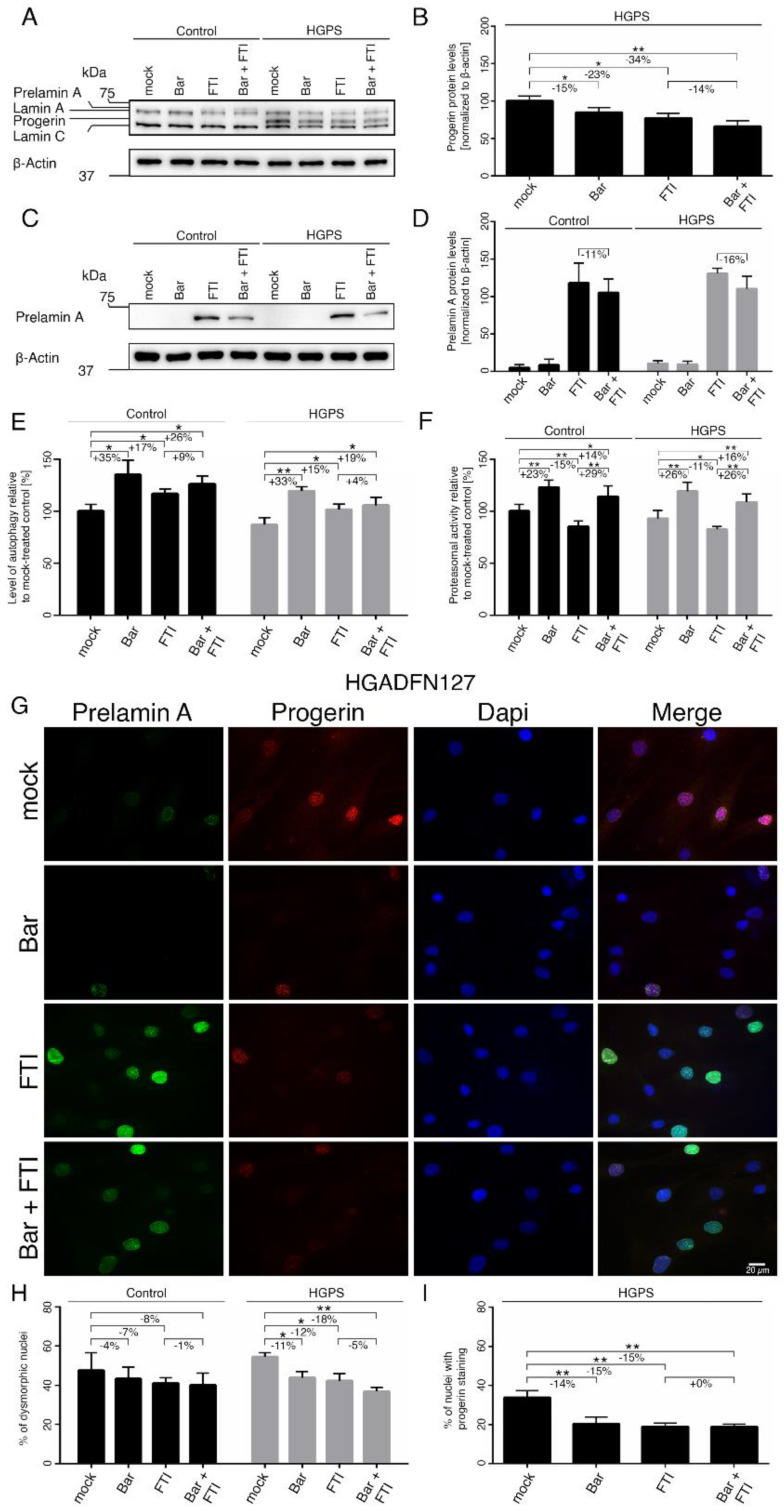
Bar, FTI and combination treatment prevent nuclear blebbing and progerin nuclear accumulation. (**A**,**C**) Representative images of western blots for lamin A/C and prelamin A. Cultures at 15% SNS were either treated with vehicle (DMSO), Bar (1 µM), FTI (0.025 µM), or combined drugs for a period of nine days. Quantification of progerin (**B**) and prelamin A (**D**). (**E**) Autophagy activity was determined by measuring MDC levels using fluorescence photometry. (**F**) Proteasome activity was determined by measuring chymotrypsin-like proteasome activity using Suc-LLVY-AMC as a substrate. (**G**) Representative immunofluorescence images of HGPS (HGADFN127) fibroblasts treated for nine days as indicated. Antibodies against prelamin A (green) and progerin (red) were used, and DNA was stained with DAPI. Fluorescence images were taken at 40× magnification. Scale bar: 20 µm. (**H**,**I**) The same staining as in (**G**) and Figure S1a was used to determine the frequency of misshapen nuclei (dysmorphic) and the number of nuclei with bright progerin signals. An average of at least 900 nuclei were counted. Graphs show mean ± SD. Representative images are shown (*n* = 3; * *p* < 0.05, ** *p* < 0.01).

To further assess the biological effect of the combination treatment, we quantified progerin levels by western blot analysis using an anti-lamin A/C antibody (Figure 4A,B).

As expected, progerin was not detected in the control fibroblasts (Figure 4A). In HGPS cells, progerin levels were reduced by Bar (−15%), FTI (−23%), and combination treatment (−34%) relative to the mock-treated counterparts (Figure 4B). The combination regimen was more effective in reducing progerin, indicating that these two compounds synergistically enhanced progerin clearance. Moreover, it is known that FTI treatment induces prelamin A accumulation in both normal and HGPS cells (Figure 4C,D). Combination treatment showed a decrease tendency in prelamin A levels (Ctrl: −11% ns, HGPS: −16% ns), indicating that Bar prevented to a certain degree, the induced prelamin A accumulation by FTI. Previous studies have established that progerin is degraded by autophagy [23,50]. Therefore, we determined the levels of autophagy in cells treated with Bar, FTI and combination treatment (Figure 4E). Bar increased autophagy levels the most (Ctrl: +35%, HGPS: +33%), FTI showed a moderate activation (Ctrl: +17%, HGPS: +15%), and combination treatment showed an intermediate increase relative to drugs alone (Ctrl: +26%, HGPS: +19%) (Figure 4E). These findings indicated that each drug induced autophagy activity; however, when combined, their effects were not additive, even though they showed a synergistic effect on the clearance of progerin in HGPS cells (Figure 4B). Next, we assessed the proteasome activity, the second degradation system of the cell. Proteasomal activity was increased by Bar (Ctrl: +23%, HGPS: +26%) and combined drugs (Ctrl: +14%, HGPS: +16%), while its activity was reduced by FTI in both cell types (Ctrl: −15%, HGPS: −11%) (Figure 4F). This indicated that FTI treatment suppressed proteasomal activity.

Nuclear envelope alterations, including invagination and blebbing, are hallmarks of HGPS cells and normal senescent cells [51,52]. Previous studies have shown that treatment with either Bar or FTI can ameliorate nuclear envelope abnormalities [7,13]. To further investigate the impact of combination treatment on nuclear envelope morphology, we analyzed progerin and prelamin A distribution (Figure 4G–I and Figure S1a). The numbers of HGPS dysmorphic nuclei were significantly reduced with Bar (−11%), FTI (−12%) and combination treatment (−18%) compared to mock-counterparts (Figure 4H). In agreement with the enhanced progerin clearance following all regimens, the number of dysmorphic and bright progerin-positive nuclei was reduced in HGPS cultures (Bar: −14%, FTI: −15%, Bar + FTI: −15%) compared to mock-counterparts (Figure 4I).

Previous studies have shown that progerin accumulation occurs concurrently with the accumulation of the linker of the nucleoskeleton and cytoskeleton (LINC) complex SUN1 at the nuclear envelope [53]. Therefore, we evaluated the status of SUN1 after all regimens (Figure S1b,c). As previously reported, SUN1 was increased in HGPS cells compared to controls (Figure S1b,c) [52,53]. FTI treatment induced an enhanced clearance of SUN1 in both cell types as the protein levels were reduced (Ctrl: −27%, HGPS: −34%) compared to mock-counterparts. On the other hand, Bar treatment did not change SUN1 levels (Figure S1b,c). Combination treatment induced a similar reduction of SUN1 in both cell types (Ctrl: −31%, HGPS: −38%) (Figure S1b,c). Collectively, combination treatment decreased the levels of progerin, prelamin A, and SUN1, thereby contributing to the normalization of the nuclear envelope morphology.

### 2.5. FTI with Bar Combination Treatment Decreases DNA Damage Levels in HGPS Cells

**Figure 5:**
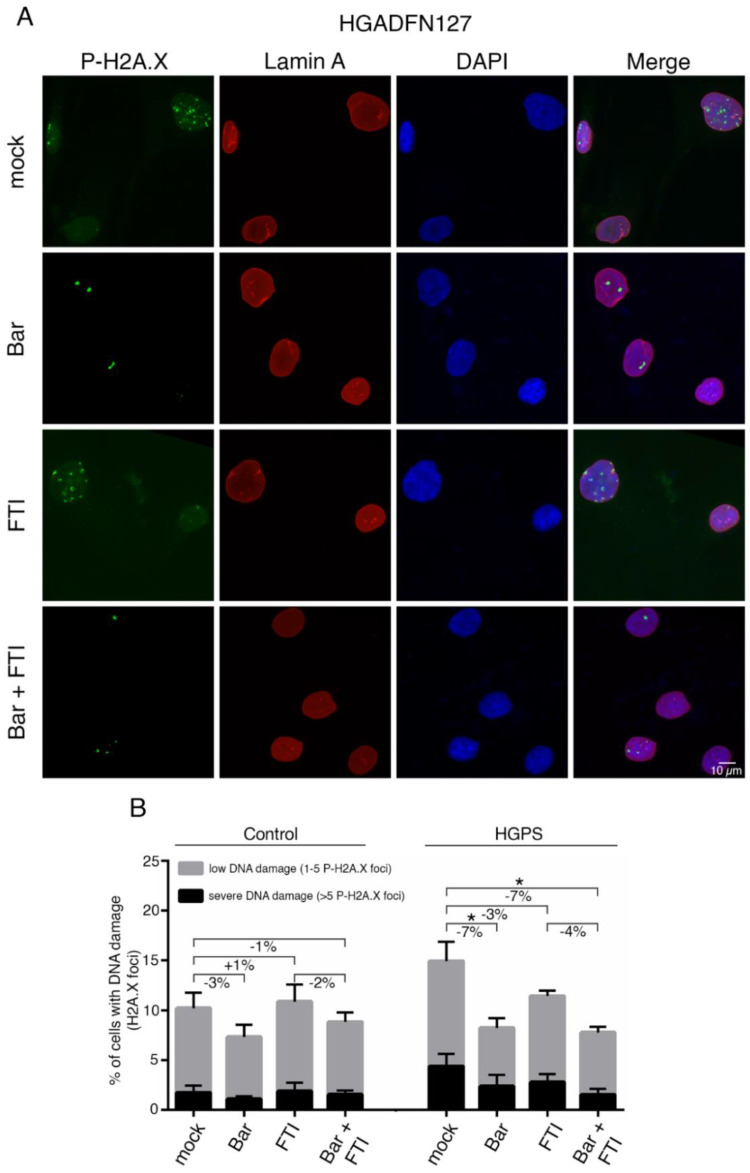
Bar and FTI combination treatment reduce P-H2A.X levels in HGPS cells. (**A**) Representative immunofluorescence images of HGPS HGADFN127 fibroblasts treated for nine days as indicated. Antibodies against P-H2A.X (green) and lamin A (red) were used, and DNA was stained with DAPI. Fluorescence images were taken at 60× magnification. Scale bar: 10 µm. (**B**) Number of nuclei with low DNA damage (1–5 P-H2A.X foci) and severe DNA damage (>5 P-H2A.X foci) in control and HGPS cultures treated as indicated. Graphs show mean ± SD. (*n* = 3; * *p* < 0.05).

Increased DNA damage is another hallmark of HGPS cells [54]. At sites of damage, several DNA damage response factors are recruited to form repair foci and can be detected using antibodies against phosphorylated γ-H2AX on Ser139 by indirect immunofluorescence (Figure 5 and Figure S2) [55].

We examined the extent to which Bar, FTI or a combination of both could improve DNA damage levels in HGPS cells (Figure 5A,B). For this, control and HGPS cultures at 15% SNS were treated with all regimens for nine days, and the number of positive γ-H2AX Ser139 nuclei was scored (Figure 5 and Figure S2). HGPS cells treated with Bar alone and in combination with FTI showed reduced levels of DNA damage (Ctrl.: −7%, HGPS: −7%) compared to mock cells, while treatment with FTI alone induced no obvious changes (Figure 5B). All treatment regimens induced no changes in control cells (Figure 5B). Collectively, FTI with Bar combination treatment reduced the frequency of γ-H2AX Ser139 foci and therefore prevented DNA damage accumulation in HGPS cells.

### 2.6. FTI with Bar Combination Treatment Increases Mitochondrial Spare Respiration Capacity and Cellular ATP Levels

**Figure 6:**
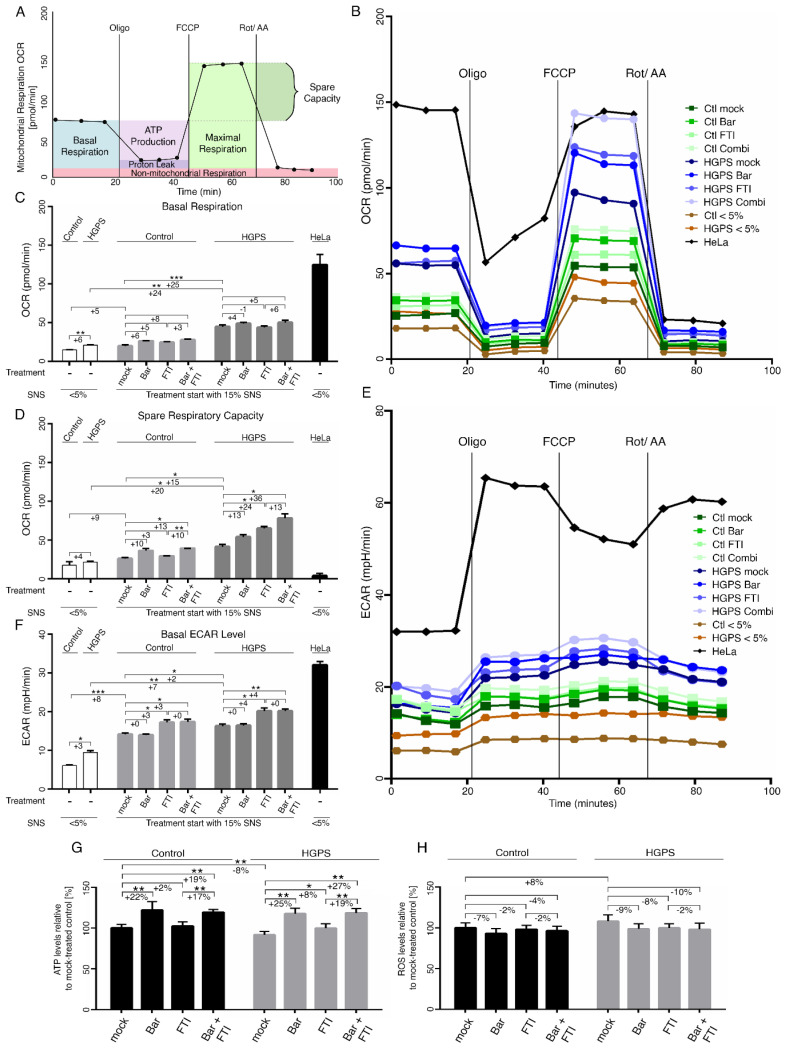
Mitochondrial function and glycolysis are impaired in HGPS fibroblasts. (**A**) Schematic representation of Seahorse XF Cell Mito stress test and calculated values are indicated. Oxygen consumption rates (OCR) (**B**) and extracellular acidification (ECAR) (**E**) were determined with a Seahorse XF96 Flux analyzer in basal and stimulated conditions (*n* = 3). Additional parameters like basal respiration (**C**), spare respiratory capacity (**D**), and basal ECAR levels (**F**) were calculated with Wave software v2.6.1.53 (Agilent Technologies, Santa Clara, CA, USA). (**G**) Cellular ATP levels were measured using a CellTiter-Glo luminescence ATP assay. (**H**) Intracellular ROS levels were determined by measuring oxidized dichlorofluorescein (DCF) levels using a DCFDA cellular ROS detection assay (*n* = 3). Graphs show mean ± SD. (* *p* < 0.05, ** *p* < 0.01, *** *p* < 0.001).

Mitochondrial dysfunction is a hallmark of HGPS cells [22,56,57]. Mitochondrial oxidative phosphorylation (OxPhos) is the primary source of ATP synthesis; however, if ATP demand is not met, premature cellular SNS or death can occur [58]. Therefore, we investigated the mitochondrial functions using a mitochondrial stress test on an XF96 seahorse analyzer. Briefly, this assay determines the mitochondrial oxygen consumption rate (OCR) from several parameters in intact cells, allowing the identification of the critical respiratory defects, as schematically indicated in Figure 6A.

Oligomycin, carbonyl cyanide-p-(tri-fluromethoxy) phenyl-hydrazone (FCCP), and rotenone/antimycin A were sequentially added to the cells to determine their ATP production, proton leak, maximal respiration, and non-mitochondrial respiration, respectively (Figure 6A). Following basal respiration, oligomycin is injected first and inhibits the ATP synthase. This affects electron propagation and leads to reduction in OCR. Next, FCCP acts as an uncoupling agent that disrupts the mitochondrial membrane potential. As a result, electron flow is not inhibited, raising OCR to the maximum. Lastly, antimycin A and rotenone inhibit electron transport chain complexes I and III, thereby completely shutting down mitochondrial respiration (Figure 6A).

Spare respiratory capacity is the difference between maximal and basal respiratory capacity and corresponds to the amount of extra ATP that can be produced by OxPhos in case of a sudden increase in energy demand [59].

The cancer cell line HeLa, which is known to rely on glycolysis to meet its energy demand, was included in this assay [60]. In accordance with previous studies, HeLa cells showed that their basal OxPhos ran at its maximal capacity, as indicated by the exhausted spare respiratory capacity (Figure 6D).

Basal and maximal respiration rates in young HGPS cells (SNS < 5%) were higher than in young controls (Figure 6B,C and Figure S3a). Moreover, basal and maximal respiration rates were also increased during replicative SNS (SNS ~15%) in both cell types. However, both of these values were consistently higher in HGPS cells (Figure 6B,C and Figure S3a). While normal cells showed no obvious changes in spare respiratory capacity during replicative SNS, HGPS cells showed an increase in this activity (Figure 6D). These data indicate that HGPS mitochondria are more oxidative and develop an adaptation response to meet their energy demands. This change in mitochondrial oxidative capacity already occurred in young HGPS cultures (<5% SNS) and, therefore, precedes the appearance of proliferation defects reported in later passages [52]. Next, we evaluated the effect of FTI and Bar, on OCR and none of these regimens induced significant changes in basal respiration rate in both control and HGPS cells (Figure 6C). However, the maximal respiration rate and spare capacity were increased by combination treatment in both cell types (Figure 6D and Figure S3a). These data indicated that FTI with Bar combination treatment increased the mitochondrial OxPhos capacity. Furthermore, the calculated ATP synthesis after oligomycin treatment showed higher ATP production in young HGPS cells compared to young control cells, and this difference further increased during replicative SNS (Figure S3b). Proton leakage was slightly increased in HGPS cells during replicative SNS, but remained low in both normal and HGPS cells (Figure S3c). None of the regimens had any effect on ATP synthesis in either cell type (Figure S3b). Collectively, FTI and Bar combination treatment improved the spare respiration capacity in HGPS cells but did not increase mitochondrial ATP production (Figure 6 and Figure S3).

Previous studies have indicated that various cell types gradually shift toward a more glycolytic state during replicative SNS [61,62]. Similarly, HGPS fibroblasts were also shown to exhibit increased glycolysis compared to normal cells [22,63]. We verified this observation by monitoring the extracellular acidification rate (ECAR) (Figure 6E). HeLa cells were used as an internal positive control for glycolysis and showed elevated ECAR levels (Figure 6E,F). ECAR analyses showed that young HGPS cultures already exhibited glycolytic behavior compared to normal cells, and this activity increased during replicative SNS in both cell types, but consistently remained higher in HGPS cells (Figure 6F). Treatment with all regimens indicated that Bar had no effect on basal glycolysis levels, but FTI induced a moderate increase in both cell types (Figure 6F). These findings indicated that FTI treatment induced a metabolic shift towards glycolysis that was not prevented by the addition of Bar in the combined treatment (Figure 6F).

Since mitochondrial OxPhos and glycolysis were increased in HGPS cells, we also determined the total intracellular ATP and ROS levels (Figure 6G,H). None of the treatment regimens resulted in a significant decrease in ROS levels in HGPS cells (Figure 6H). While ATP levels were significantly reduced in HGPS cells relative to normal cells, Bar with or without FTI treatment increased the levels of ATP in both cell types (Figure 6G). These results indicated that FTI, despite inducing glycolysis, showed less efficacy in restoring ATP levels as observed with Bar alone.

ECAR revealed a shift to glycolysis in both HGPS and normal cells during replicative SNS, and this switch worsened upon FTI treatment (Figure 6E,F). Combining the OCR and ECAR data in a bioenergetic profile (Figure S3d) clearly demonstrates that both activities increased during replicative SNS and this tendency was greater in HGPS cells (Figure S3d). Cells treated with both drugs showed an additive metabolic shift toward glycolysis and OxPhos associated with FTI and Bar, respectively. This contributed to ameliorating the energy profile of the cells, as indicated by the increased intracellular ATP levels in both cell types. Collectively, HGPS cells exhibit early metabolic changes, resulting in increased OCR and ECAR relative to normal cells. FTI and Bar combination treatment improved mitochondrial spare-respiratory capacity and basal ECAR levels, resulting in the increased intracellular ATP levels.

## 3. Discussion

Today, lonafarnib is the only FDA-approved treatment for children with HGPS, but still not a cure [36]. The clinical trial of lonafarnib demonstrated significant amelioration in some aspects of the disease development, including increased rate of weight gain and improved measures of cardiovascular stiffness, bone structure, and hearing [31]. Even though FTI (lonafarnib) treatment is associated with several mild side effects, including nausea, vomiting, diarrhea, and fatigue, it has overall good tolerability and safety [64]. However, at the cellular level, FTI induces mitotic defects, leading to genomic instability and donut-shaped nuclei [25]. Consequently, to improve the prognosis of lonafarnib treatment for children with HGPS, compounds abrogating FTI-negative cellular effects and ameliorating cell functions that are not restored by FTI are needed.

This study tested the extent to which FTI combined with a JAK-STAT inhibitor (Bar) could restore the HGPS cellular phenotype compared to single drugs alone. This drug combination was effective in lowering cellular inflammation and enhancing progerin clearance in HGPS cells. Furthermore, decreased progerin content ameliorated nuclear morphology; regardless, DNA damage and mitochondrial function were only partially improved. While Bar reduced DNA damage levels in HGPS cells, FTI induced a high frequency of cytoplasmic chromatin foci formation, which can be considered as a form of extranuclear DNA damage [65].

Several molecular mechanisms might converge into the generation of these extranuclear DNA foci in cells treated with FTI. Indeed, besides affecting prelamin A processing, FTI can also act on several other farnesylated proteins that are critical for the cell cycle progression. For instance, FTI can block the farnesylation of two centromere-associated kinetochore proteins, namely CENP-E and CENP-F [66]. CENP-E is a kinesin motor protein required for establishing the kinetochore-microtubule connection, allowing the segregation of the sister chromatids during mitosis [67], while CENP-F is temporarily recruited to kinetochores during prometaphase/metaphase transition [68]. Therefore, FTI-induced mitotic defects could account for the presence of cytoplasmic chromatin fragments at the end of mitosis. Furthermore, FTI can affect normal lamin B processing, which leads to the depletion of lamin B1 from the nuclear envelope and the delocalization of lamin B2 to the nucleoplasm [35]. In this context, accumulation of unfarnesylated lamin B would lead to severe alterations in the nuclear lamina composition that would compromise the integrity of the nuclear envelope [35]. Consequently, these defects in the nuclear envelope architecture could allow nuclear blebbing and the generation of cytosolic DNA fragments. In this study, we showed that FTI induced the same frequency of cytosolic DNA foci in both normal and HGPS cells, indicating that this effect is directly linked to FTI activity and not to progerin expression.

Several studies have demonstrated that extranuclear DNA foci can activate the cGAS-STING signaling pathway [65,69]. cGAS is a cytosolic DNA sensor that is activated after recognition of either pathogenic and self-DNA and then generates cyclic GMP-AMP (cGAMP), which acts as a second messenger that stimulates STING, leading to two downstream pathways: type I interferon through interferon regulatory factor 3 (IRF3) and pro-inflammatory response through NF-κB [70,71,72].

In accordance with the activation of cGAS-STING, FTI-treated cultures showed elevated levels of IFN-β, which caused activation of JAK-STAT signaling cascades, as indicated by the increased levels of p-STAT1. Importantly, cells treated with Bar (JAK-STAT inhibitor) showed barely detectable levels of activated STAT1 (p-STAT1) even in the presence of FTI. These findings demonstrated that Bar treatment could dampen the cGAS-STING signaling elicited by FTI. The ability of Bar to correct this FTI side effect might underlie other ameliorations of the HGPS phenotype, including increased cell proliferation and delayed SNS.

Previous studies have implicated the dysregulation of two inflammatory pathways in HGPS disease, namely the NF-ƙB- and JAK-STAT pathways [7,42,73,74]. While inflammation is normally induced during infections or tissue injuries, accumulation of senescent cells can also trigger low-grade inflammation in tissues [75]. Activation of NF-ƙB and JAK-STAT occurs during replicative SNS, and is accompanied by a robust increase in the secretion of numerous cytokines, chemokines, growth factors, and extracellular matrix protease [7]. Together, these factors are termed SASPs [76]. Moreover, distinct SASP molecules including IFN-β, IL-1α, CCL2, IL-6, and CXCL8 have been implicated in sustaining chronic inflammation called “inflammaging” [77]. These factors can directly modulate the immune response, but become destructive to tissues and organs when inflammation persists [78]. Remarkably, Bar treatment was effective in reducing the expression levels of all the cytokines mentioned above and this to the same extent when Bar was combined with FTI.

Furthermore, emerging evidence implicates cGAS-STING as a crucial regulator of SNS and the SASP in normal cells exposed to various stresses [65]. By analogy, FTI treatment causes mitotic stress leading to cGAS-STING signaling, which might explain the dose-dependent FTI decrease in cell growth and promotion of SNS as observed in different cell types including cancer cells [79,80]. The fact that Bar showed efficacy in inhibiting FTI-induced cGAS-STING-STAT1 activation further supports the assumption that combination treatment might delay activation of this signaling axis triggered by the processes inherent to normal cellular aging. In this context, Bar-FTI combination treatment might show promise in decreasing inflammation and aging-associated inflammation.

In addition to DNA damage, which is considered an important trigger of SNS, mitochondrial dysfunction is also a crucial contributor. Previous studies have indicated that senescent cells are dependent on both glycolysis and OxPhos to maintain cellular functionality; however, glycolysis is increased to compensate for mitochondrial dysregulation [22,81,82]. Age-related malfunctions, such as misfolding of proteins, are associated with high energy demand, as the process of protein degradation requires high levels of ATP [83]. We determined the oxygen consumption rates to measure OxPhos and ECAR as an indicator of glycolysis and found that both activities were increased during replicative SNS of both normal and HGPS cells. However, HGPS cells, exhibited a higher OxPhos and glycolysis levels in early passages (~5% SNS) compared to matched normal cells. This observation indicated that HGPS cells due to progerin expression enhanced glycolysis to meet their energy demands. This suggests that young HGPS cells develop a form of metabolic adaptation in response to the cellular stress induced by progerin expression. Previous metabolic studies on HGPS mouse and human fibroblasts reported that glycolysis was increased compared to that in control fibroblasts [22,63,81]. However, these studies did not consider the SNS index, rate of oxygen consumption, or other parameters of the respiratory chain. Nevertheless, all these studies are in agreement with the increased glycolysis in HGPS cells. Evaluating the effect of Bar and FTI on OxPhos and glycolysis showed that these drugs acted differently. Bar increased OxPhos, while FTI increased glycolysis in both normal and HGPS cells. Combined treatment raised both activities to the levels reached by single drugs and showed no additive effect on either of these functions.

Although none of the drugs showed significant changes in basal respiration rate, the maximal respiration rate and spare capacity were increased by Bar with or without FTI. These data indicate an increased mitochondrial OxPhos capacity, which is important when higher energy expenditure is required to maintain cellular homeostasis. The basal ECAR levels revealed that FTI increased glycolysis while Bar had no effect, and consequently, combination treatment raised the glycolysis to the same levels as FTI alone.

The outcome of these treatments on total cellular ROS and ATP levels indicated that Bar increased ATP levels, but had no significant effect on ROS. In contrast, FTI induced no significant changes on either parameter. Combination treatment induced similar outcomes to Bar in both normal and HGPS cells. Since ROS levels were not reduced by either drug, these findings suggest that mitochondrial function was not rescued and future investigations will be needed to identify the causes. The fact that total cellular ATP was raised by Bar and combination treatment might have further positive outcomes in patients with HGPS. Indeed, previous studies have shown that both children with HGPS and HGPS mouse models have decreased levels of extracellular pyrophosphate, a regulator of calcification that leads to characteristic vascular calcifications [84,85,86]. Since ATP hydrolysis is the main source of pyrophosphate, treatment of HGPS mice with ATP could prevent vascular calcification [85]. While further investigations are needed, the observation that Bar treatment of HGPS cells with or without FTI could ameliorate ATP levels a potential improvement in pyrophosphate levels could be expected, which would reduce the vascular stiffness affecting patients with HGPS.

In this study, we provide evidence that FTI induces mitotic defects, resulting in genomic instability with donut-shaped nuclei and an increase in extranuclear DNA fragments. These defects were associated with reduced proliferation and activation of the cGAS-STING-STAT1 signaling axis, increased expression of SASPs, and a metabolic shift towards glycolysis. When Bar was co-administrated with FTI, it significantly reduced some of the cellular side effects of FTI. Bar and FTI co-treatment ameliorated cell growth, inhibited the cGAS-STING-STAT1 pathway, reduced the levels of SASPs, increased OCR, and ameliorated total cellular ATP levels. Collectively, the ability of Bar to counteract the negative effects of FTI might contribute to delaying premature senescence of HGPS cells. This would in turn reduce the levels of inflammation known to significantly impact the progression of several symptoms developed by children with HGPS.

Cellular SNS is an evolving, multistep process over time that ultimately influences numerous cellular functions that impact cellular homeostasis and, consequently, organ function [87,88]. Although further *in vivo* studies are needed to evaluate the efficacy of Bar and FTI combination treatment in an HGPS mouse model, the functional ameliorations observed in HGPS cells show promise. Moreover, following the recent FDA approval of lonafarnib, all children suffering from HGPS are medicated with lonafarnib, and further treatments should be identified to ameliorate HGPS symptoms and reduce FTI cellular side effects [4]. Bar (Olumiant^®^) is already prescribed for patients with rheumatoid arthritis, a common symptom in patients with HGPS. Hence, Bar has been investigated in numerous clinical trials, including alopecia areata (hair loss), which also develops in HGPS [89]. This study provided evidence that combining two FDA-approved drugs (lonafarnib with baricitinib) showed positive synergistic effects in human HGPS fibroblast homeostasis. The efficacy of this combination therapy needs to be further evaluated in HGPS mouse models to determine its potential value as a therapeutic strategy for children with HGPS and possibly other age-related conditions.

## 4. Materials and Methods

### 4.1. Cell Culture and Drug Treatment

All fibroblasts from patients with HGPS were supplied by the Progeria Research Foundation Cell and Tissue Bank (http://www.progeriaresearch.org, accessed on 7 July 2021. The following fibroblasts were used: HGADFN003 (2-year-old male) and HGADFN127 (3-year-old female). Control fibroblasts were supplied by the Coriell Institute for Medical Research (Camden, NJ, USA). The following cell lines were used: GM01651C (13-year-old female) and GM01652C (11-year-old female). All cells were cultured in Dulbecco’s Modified Eagle Medium (DMEM) with GlutaMAX™ (Thermo Fisher, Waltham, MA, USA), supplemented with 15% fetal bovine serum (FBS, Thermo Fisher), 1% L-glutamine (Thermo Fisher), 1% penicillin-streptomycin (10,000 U/mL; Thermo Fisher), and 0.5% gentamicin (10 mg/mL, Thermo Fisher). Passage numbers corresponding to the percentage of SNS are indicated in Table 1.

In earlier studies, we assessed the proliferation rate and cytotoxicity to determine the optimal drug concentration of Bar and FTI and found that 1 µM Bar and 0.025 µM FTI were the most effective [7,23]. Fibroblasts were either treated with Bar (Absource Diagnostics GmbH), FTI lonafarnib (Merck) or a combination of the two drugs. Mock-treated cells were treated with the vehicle (DMSO). The medium of all treatments was changed every two days. During the nine days of treatment, the cells were split once.

### 4.2. Western Blot

Fibroblasts were harvested by scraping the cell culture samples. Briefly, a total of 25 µg of isolated proteins was separated on precast protein gels (4–20% Mini-PROTEAN^®^ TGX™; Bio-Rad, Hercules, CA, USA) and transferred onto nitrocellulose membranes. To detect the proteins of interest, membranes were blocked for 1 h in 5% non-fat milk and were then incubated overnight at 4 °C with the following primary antibodies: anti-STAT1 (#14994, Cell Signaling, Danvers, MA, USA, dilution 1:1000), anti-P-STAT1 (#9167, Cell Signaling, dilution 1:1000), anti-STAT3 (#9139, Cell Signaling, dilution 1:1000), anti-P-STAT3 (#9145, Cell Signaling, dilution 1:1000), anti-lamin A/C (sc-20681, Santa Cruz Biotechnology, Dallas, TX, USA, dilution 1:10,000), anti-prelamin A (MABT858, Sigma-Aldrich, St. Louis, MO, USA, dilution 1:2000), anti-SUN1 (HPA008346, Sigma-Aldrich, dilution 1:500) and anti-ß-Actin (#A1978, Sigma-Aldrich, dilution 1:10,000). After washing in TBS Tween, the membranes were incubated for 1 h with a corresponding secondary antibody conjugated with horseradish peroxidase (Jackson ImmunoResearch Laboratories, West Grove, PA, USA). For signal amplification, luminol-enhanced chemiluminescence was performed. Staining was visualized using a ChemiDoc™ MP, and densitometry was performed using ImageJ software (NIH, software version 1.5). Blots were quantified by normalizing to ß-actin. A mild stripping buffer was used to reuse the membranes.

### 4.3. Senescence Associated Beta-Galactosidase Assay

SNS was assessed in HGPS and control cultures prior to and after treatment using the SA-ß-galactosidase protocol as described by Dimri et al. [90]. Briefly, the cells were fixed with 2% formaldehyde for 5 min and incubated at 37 °C (without CO_2_) overnight in fresh 5-bromo-4-chloro-3-indolyl-D-galactopyranoside (X-gal) solution. The staining solution contained 1 mg/mL X-gal, 40 mM citric acid/sodium phosphate (pH 6.0), 5 mM potassium ferrocyanide, 5 mM potassium ferricyanide, 150 mM NaCl, and 2 mM MgCl2. Blue cells were counted using the Cell Counter plugin from the ImageJ software. A total of 1000 cells were counted for each sample.

### 4.4. Determination of Cell Number

Before treatment, cells were seeded (1.5 × 105) in 10 cm dishes. Following drug treatment, cells were trypsinized and counted with a Muse™ Cell Analyzer (Merck Millipore, Burlington, MA, USA) using DNA-binding dyes, to assess the fluorescent signal.

### 4.5. Autophagy Activity

Autophagic activity and digestion of intracellular components were measured using Cayman’s Autophagy/Cytotoxicity Dual Staining Kit (Cayman Chemical Company, Ann Arbor, MI, USA). The kit employs monodansylcadaverine (MDC), an autofluorescent substance incorporated into multilamellar bodies used for the detection of autophagic vacuoles in cultured cells. Cells were seeded (3.2 × 104) in quintets in 96-well plates and were allowed to attach overnight. MDC was added to the wells at a 1:100 ratio. Measurements of the autophagic vacuoles intensities were performed using a FLUOstar Omega Microplate Reader (BMG Labtech, Ortenberg, Germany), with an excitation wavelength of 355 nm and an emission wavelength of 520 nm. All measurements were performed in at least three independent experiments.

### 4.6. Measurement of ROS

Cells were seeded (3.2 × 104) in 96-well plates and were allowed to attach overnight. The cellular ROS detection assay kit (Abcam) was used to measure the amount of ROS. After attachment, cells were incubated with 25 µM 2′,7′-dichlorofluorescein diacetate (DCFDA) for 45 min at 37 °C. DCFDA diffuses across the cell membrane, deacetylates to form H2DCF, and then reacts with intracellular ROS to form DCF, which is a fluorescent dye that monitors real-time ROS. Fluorescence was measured using a FLUOstar Omega Microplate Reader (BMG Labtech) at the excitation and emission wavelengths of 485 nm and 520 nm, respectively, after a washing step. All measurements were performed in at least three independent experiments.

### 4.7. Proteasomal Activity

The 20S Proteasome Assay Kit (Cayman Chemical Company) was used to determine proteasomal activity in cell cultures. Briefly, the kit employs a specific 20S substrate that generates a highly fluorescent product upon cleavage by the active enzyme. An equal number of cells were seeded (3.2 × 104) for each sample and the assay was performed according to the manufacturer’s instructions. The fluorescent signal was measured using a FLUOstar Omega Microplate Reader (BMG Labtech) at an excitation wavelength of 485 nm and an emission wavelength of 520 nm. All measurements were performed in triplicates in at least three independent experiments.

### 4.8. Measurement of Intracellular ATP

The amount of ATP was measured using the CellTiter-Glo^®^ luminescent cell viability assay (Promega, Madison, WI, USA). After cell lysis, the amount of ATP present in the culture was determined by assessing the luminescent signal generated during the luciferase reaction. An equal number of cells were seeded (3.2 × 104) in quintets in 96-well plates. After 12 h of seeding, cells were incubated with the CellTiter-Glo reagent for 10 min, and the luminescence intensity measured using a FLUOstar Omega Microplate Reader (BMG Labtech). The ATP standards were assessed in parallel. All measurements were performed in at least three independent experiments.

### 4.9. Respirometric Assay

OCR and ECAR measurements in intact fibroblast cells were analyzed using a Mito Stress Test Kit and an XF96 Seahorse Analyzer (Agilent Technologies), according to the manufacturer’s protocol. We seeded 1.5 × 104 fibroblasts into each well of the 96-well plate and used three technical replicates for each condition. Preparation of the Seahorse XF DMEM assay medium included 10 mM glucose, 1 mM pyruvate, and 2 mM L-Glutamine. For the injection of the components, drugs were diluted to a 10× solution to get a final working concentration of 2.5 µM oligomycin, 2 µM Carbonyl cyanide-4 (trifluoromethoxy) phenylhydrazone (FCCP), and 1 µM rotenone/antimycin A. The injection ports of the flux cartridge were loaded with the defined volumes of the respective compounds. Oligomycin (20 µL) was applied to ports A, 22 µL FCCP was pipetted into ports B and 25 µL rotenone/antimycin A was added to ports C.

### 4.10. Immunocytochemistry

Fibroblasts in culture were seeded on coverslips and subsequently fixed with ice-cold 100% methanol at −20 °C for 10 min. Once fixated, the cells were blocked at 25 °C for 30 min in 10% FBS in PBS-T (0.2% Tween) and then incubated with the following primary antibodies: anti-p21 (MA5-14949, Invitrogen, Waltham, MA, USA, dilution 1:250), anti-lamin A/C (#E-1 sc-376248, Santa Cruz, dilution 1:2500), anti-cGAS (#15102, Cell Signaling, dilution 1:100), anti-progerin [56], anti-prelamin A (MABT858, Sigma-Aldrich, dilution 1:250), anti-P-H2A.X Ser139 (#05-636, Sigma-Aldrich, dilution 1:2000), and anti-lamin A (#L1293, Sigma-Aldrich, dilution 1:2000). After washing with PBS-T, the samples were incubated with the corresponding secondary antibodies at 25 °C for 1 h: affinity-purified Alexa Fluor 488 goat or donkey IgG antibodies (Life Technologies, Carlsbad, CA, USA) and Cy3-conjugated IgG antibodies (Jackson ImmunoResearch). All samples were counterstained with DAPI in Vectashield mounting medium (Vector Inc., Burlingame, CA, USA). Images were acquired using an Axio Imager D2 fluorescence microscope (AxioCam MRm, Objective 40× oil NA 1.4; Carl Zeiss, Oberkochen, Germany). For statistical evaluation, 800 to 1000 cells were screened in 3 independent experiments.

### 4.11. Gene Expression Analysis

RNA was extracted from the cell pellets using the GeneJET RNA Purification Kit (Thermo Fisher Scientific Inc.). Dermal fibroblasts (~1 × 106) were collected for RNA preparation. RNA quantity and purity were assessed using a NanoDrop spectrophotometer (NanoDrop ND-1000, Thermo Fisher). In total, 1000 ng of RNA was reverse-transcribed into cDNA, using a High-Capacity cDNA Reverse Transcription Kit (Thermo Fisher). Real-time PCR primers were designed by using NCBI/Primer-BLAST [91]. All the primers were purchased from Thermo Fisher. All the evaluated genes and their corresponding primers are listed in the primer list (Table S1). Real-time PCR was performed using the PowerUp^TM^ SYBR™ Green Master Mix (Applied Biosystems™, Thermo Fisher) in a StepOnePlus™ Real-Time PCR System (Thermo Fisher). For optimal detection, we used 300 nM of each primer and 50 ng of the template in a 20 µL reaction volume. The thermal profile consisted of an initial denaturation step at 95 °C for 20 s, followed by 45 cycles of 95 °C for 3 s and 60 °C for 30 s. Amplification signals were all observed between cycles 10 and 40. All experiments were performed in at least three replicates and at least three independent experiments. GAPDH was used as an endogenous control.

### 4.12. Statistical Analysis

Comparative analysis of different characteristics of HGPS and healthy control cells was conducted using the Student’s *t*-test. mRNA content was calculated by using a pairwise fixed reallocation randomization test [92]. (*n* ≥ 3, * *p* < 0.05, ** *p* < 0.01, *** *p* < 0.001). *p* < 0.05 was considered to be statistically significant. Values are presented as mean ± standard deviation (mean ± SD). All statistical analyses were performed using the GraphPad Prism software version 6.01 (San Diego, CA, USA).

## Figures and Tables

**Table 1:** Passage numbers for each cell line corresponding to the indicated senescence index (SNS).

Cell Line	≤5% SNS (0–5%)	~13–17% SNS
1651C	Passage ≤ 21	Passage 26–28
1652C	Passage ≤ 20	Passage 26–27
P003	Passage ≤ 17	Passage 21–22
P127	Passage ≤ 17	Passage 22–23

## Data Availability

Data are contained within the article or Supplementary Materials.

## Supplementary Materials

The following are available online at https://www.mdpi.com/article/10.3390/ijms22147474/s1.
