# Supplementary material for: Baricitinib, a JAK-STAT Inhibitor, Reduces the Cellular Toxicity of the Farnesyltransferase Inhibitor Lonafarnib in Progeria Cells
Source: Int J Mol Sci. 2021 Jul 12;22(14):7474. doi: 10.3390/ijms22147474 (PMC8307450; doi:10.3390/ijms22147474)
Supplement: Supplementary file 1 [file ijms-22-07474-s001.zip › Supplementary.pdf]

International Journal of
Molecular Sciences

MDPI

Baricitinib, a JAK-STAT inhibitor, reduces the cellular toxicity of the farnesyltransferase inhibitor lonafarnib in progeria cells

Rouven Arnold [1], Elena Vehns [1], Hannah Randl [1], and Karima Djabali [1,*]

[1] Epigenetics of Aging, Department of Dermatology and Allergy, TUM School of Medicine, Technical University of Munich (TUM), 85748 Garching, Germany; rouven.arnold@tum.de (R.A.); elena.vehns@tum.de (E.V.); hannah.randl@tum.de (H.R.)
* Correspondence: djabali@tum.de; Tel.: +49-89-289-10920

Supplementary Figures

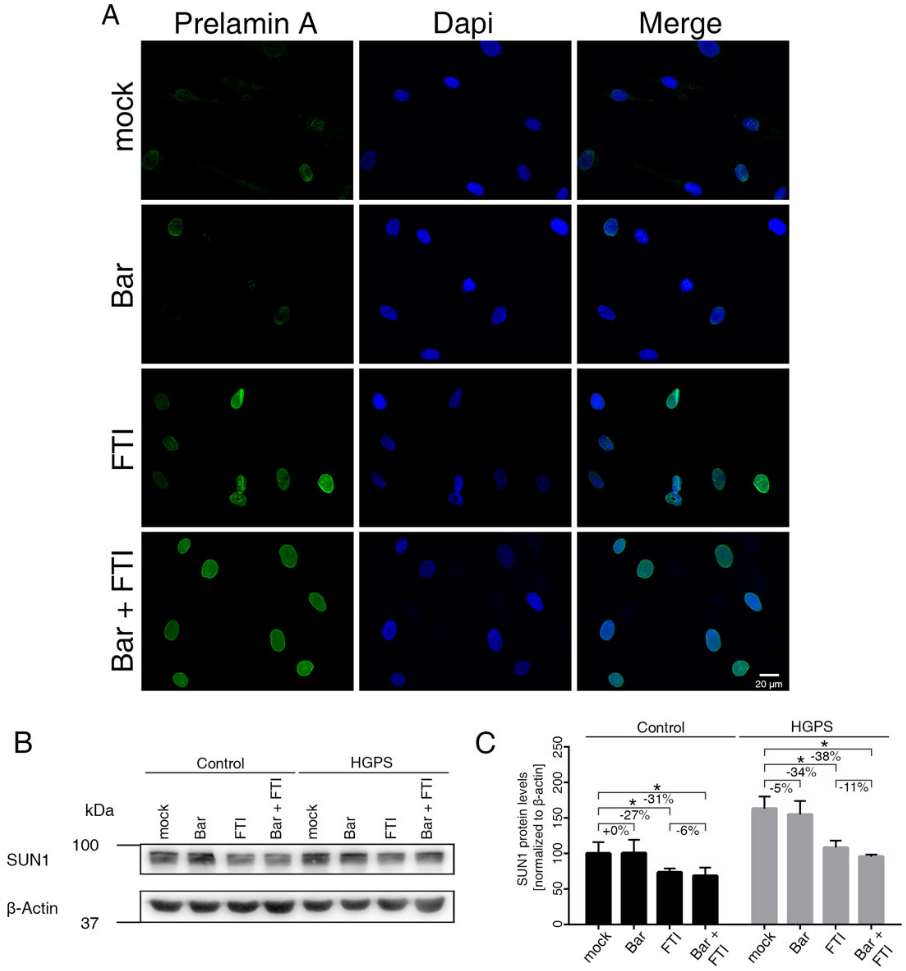

Figure S1. Bar and FTI combination treatment prevents nuclear blebbing in control cells and reduces SUN1 levels. (a) Representative immunofluorescence images of control (GM01651C) fibroblasts treated for nine days as indicated. Antibody against prelamin A (green) was used, and DNA was stained with DAPI. Fluorescence images were taken at 40x magnification. Scale bar 20 µm. (b) Representative images of western blots for SUN1 and (c) quantification of SUN1. Graphs show mean ± SD. Representative images are shown (n=3; *$p < 0.05$)

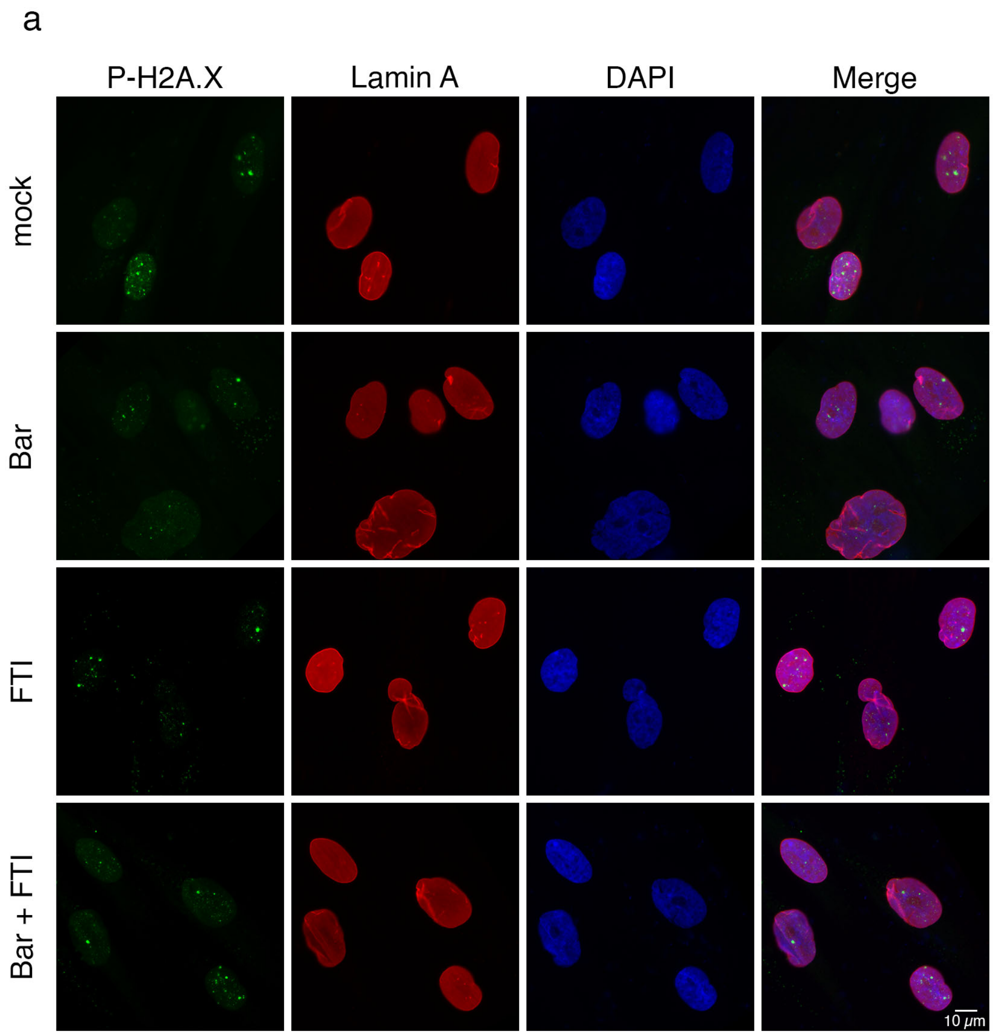

Figure S2. Bar and FTI combination treatment induces no changes in P-H2A.X levels in control cells. (a) Representative immunofluorescence images of control GM01651C fibroblasts treated for nine days as indicated. Antibodies against P-H2A.X (green) and lamin A (red) were used, DNA was stained with DAPI. Fluorescence images were taken at 60x magnification. Scale bar: 10 μm.

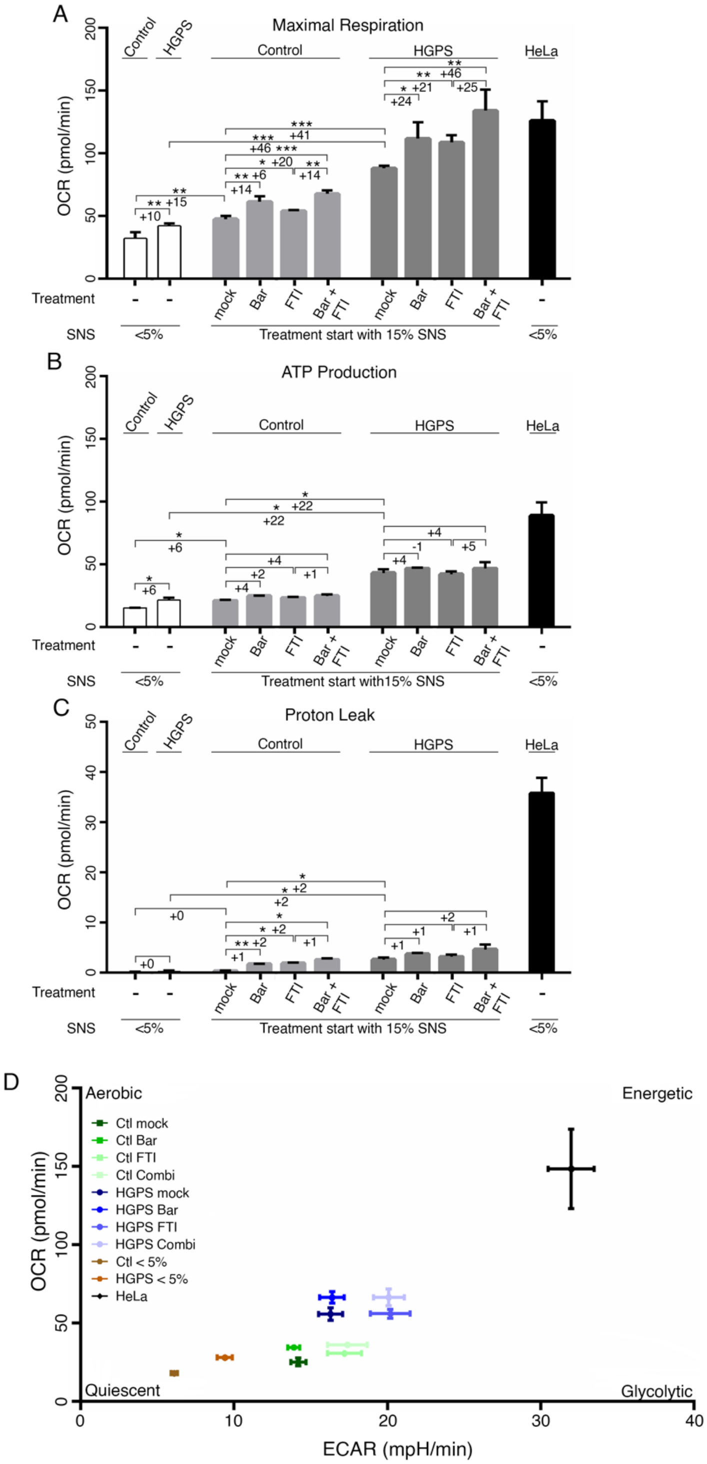

Figure S3. Mitochondrial function and glycolysis are altered in HGPS fibroblasts. Oxygen consumption rates (OCR) and extracellular acidification (ECAR) were determined with a Seahorse XF Flux Analyzer in basal and stimulated conditions (n=3). Maximal respiration (a), ATP production (b), and proton leak (c) were calculated with Wave software v2.6.1.53 (Agilent Technologies). (d) Plotting basal ECAR and OCR levels provides a snap-shot of the bioenergetics profiles. Graphs show mean ± SD. (*p < 0.05, **p < 0.01, ***p < 0.001).

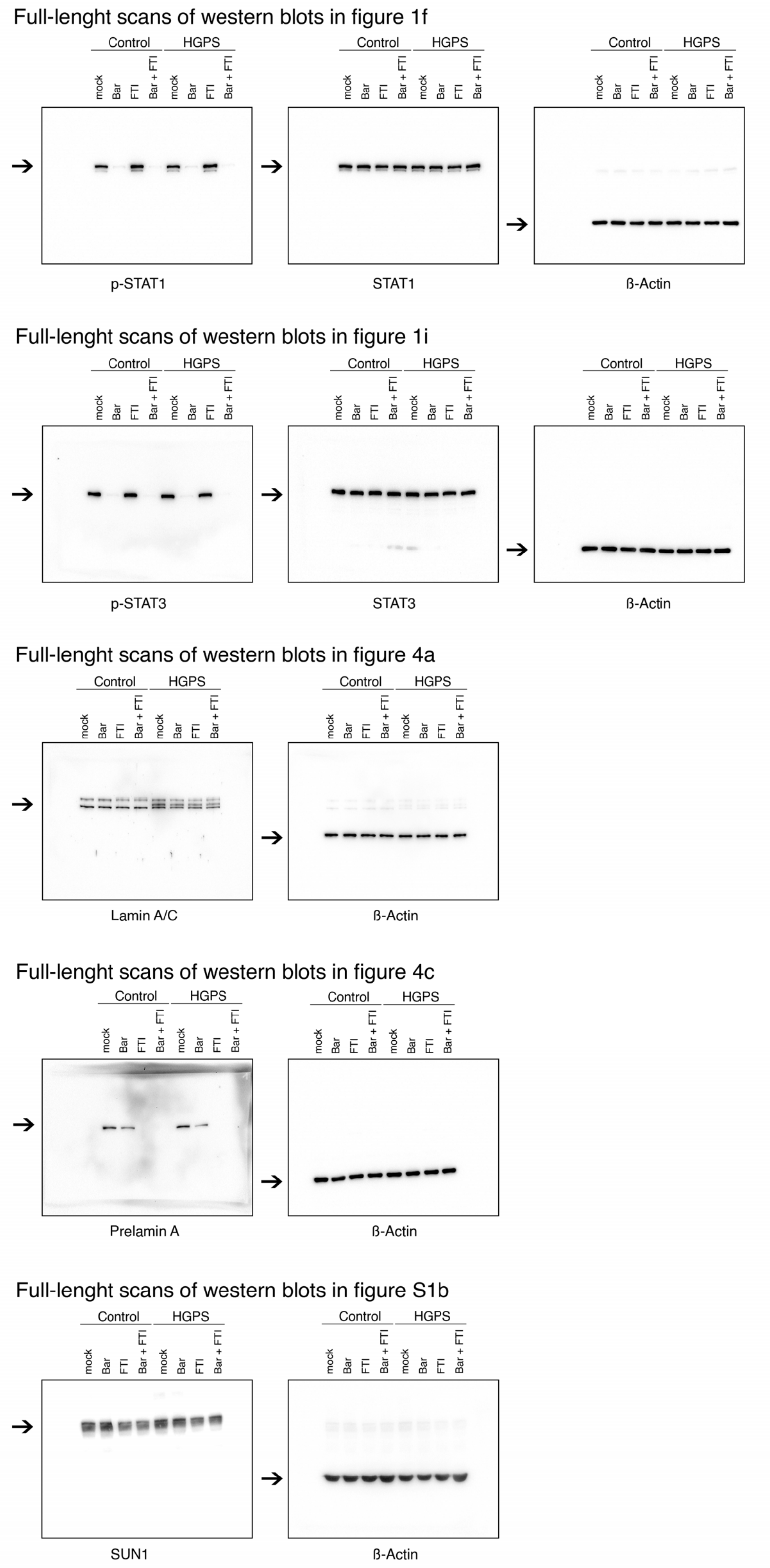

Figure S4. Full-length scan of western blots from Figures 1f, i, Figure 4a, c, Figure S1b.

Table S1. Primers used for real-time quantitative PCR analysis

Primer sequence	Target Gene	Product size (bp)	Melting Temp	Info
FW:5’-GCTTGGATTCCTACAAAGAAGCA-3’<br>REV:5’-ATAGATGGTCAATGCGGCGTC-3’	IFN-β	166	59.24 °C<br>60.87 °C	
FW:5’-CGCCAATGACTCAGAGGAAGA-3’<br>REV:5’-AGGGCGTCATTCAGGATGAA-3’	IL-1α	120	59.79 °C<br>59.09 °C	doi.org/10.1371/journal.pone.0002301
FW:5’-AGCATGAAAGTCTCTGCCGC-3’<br>REV:5’-GGCATTGATTGCATCTGGCTG-3’	CCL2	93	62.9 °C<br>66.0 °C	doi.org/10.1172/jci.insight.87023
FW:5’-GGTACATCCTCGACGGCATCT-3’<br>REV:5’-GTGCCTCTTTGCTGCTTTCAC-3’	IL-6	81	63.6 °C<br>62.4 °C	doi.org/10.1152/ajpendo.00255.2002
FW:5’-CTGGCCGTGGCTCTCTTG-3’<br>REV:5’-CCTTGGCAAAACTGCACCTT-3’	CXCL8	69	63.2 °C<br>62.5 °C	doi.org/10.1172/jci.insight.87023
